# How the threat of losses makes people *explore* more than the promise of gains

**DOI:** 10.3758/s13423-016-1158-7

**Published:** 2016-09-12

**Authors:** Tomás Lejarraga, Ralph Hertwig

**Affiliations:** Center for Adaptive Rationality, Max Planck Institute for Human Development, Lentzeallee 94, 14195 Berlin, Germany

**Keywords:** Search, Decisions from experience, Exploration, Loss attention, Loss aversion

## Abstract

Until recently, *loss aversion* has been inferred exclusively from choice asymmetries in the loss and gain domains. This study examines the impact of the prospect of losses on exploratory search in a situation in which exploration is costly. Taking advantage of the largest available data set of decisions from experience, analyses showed that most people explore payoff distributions more under the threat of a loss than under the promise of a gain. This behavioral regularity thus occurs in both costly search and cost-free search (see Lejarraga, Hertwig, & Gonzalez, *Cognition, 124*, 334–342, 2012). Furthermore, a model comparison identified the simple win-stay-lose-shift heuristic as a likely candidate mechanism behind the loss–gain exploration asymmetry observed. In contrast, models assuming loss aversion failed to reproduce the asymmetry. Moreover, the asymmetry was not simply a precursor of loss aversion but a phenomenon separate from it. These findings are consistent with the recently proposed notion of intensified vigilance in the face of potential losses.

**Electronic supplementary material:**

The online version of this article (doi:10.3758/s13423-016-1158-7) contains supplementary material, which is available to authorized users.

“Losses loom larger than gains” (p. 279) proposed Kahneman and Tversky, in their influential 1979 article. Since then, *loss aversion*—the idea that people are more concerned with losses than with gains—has been used to explain a wide range of classic behavioral regularities, such as framing effects (Tversky & Kahneman, 1981), the disposition effect (Weber & Camerer, 1998), the endowment effect (Thaler, 1980), and the sunk-cost effect (Arkes & Blumer, 1985). For decades, loss aversion was *inferred* from choice, with no account being taken of other dimensions from which the aversion to losses or its potential precursors could be gauged. It is only recently that researchers have begun to study loss aversion by reference to other accessible dimensions, including neural activation (Rick, 2011; Tom, Fox, Trepel, & Poldrack, 2007), physiological arousal (Hochman & Yechiam, 2011; Yechiam, Retzer, Telpaz, & Hochman, 2015), attention (Yechiam & Hochman, 2013a, 2013b, 2014), and exploratory search (Lejarraga, Hertwig, & Gonzalez, 2012). The last of these is the focus of this article.

## Exploratory search in decisions from experience

To what extent can traces or precursors of loss aversion be found in behavior beyond choice? To answer this question, Lejarraga et al. (2012) took advantage of a fast-growing body of research concerning the *description–experience gap*. In recent years, many investigations have sought to understand the extent to which choices between monetary gambles (payoff distributions) differ systematically when decision makers draw on firsthand experience of the probabilistic structure of those gambles (“decisions from experience”) as opposed to when they are informed about the structure of those gambles in symbolic form (“decisions from description”; for a review, see Hertwig & Erev, 2009; see also Hertwig, 2016). Many of these investigations have employed a simple tool to study decisions from experience: a “computerized money machine.” Participants see two buttons on a computer screen, each representing an unknown payoff distribution. Clicking a button results in a random draw from the specified distribution. Most studies have used two variants of this experimental tool. In the *sampling paradigm* (e.g., Hertwig, Barron, Weber, & Erev, 2004; Weber, Shafir, & Blais, 2004), participants first sample as many outcomes as they like and only then decide from which distribution to make a single draw for real. In the *partial-feedback paradigm* (e.g., Barron & Erev, 2003; Erev & Barron, 2005), in contrast, each draw contributes to participants’ earnings, and they receive draw-by-draw feedback on their obtained payoffs.

Both experienced-based experimental paradigms offer an advantage relative to the study of decisions from description. They lay open what is otherwise more difficult to discern: people’s search for information.^1^ Search behavior in the sampling paradigm is not incentivized *directly*, but only indirectly via the final choice a person makes. Let us explain this point by considering a key difference between the sampling and the partial-feedback paradigms, namely, the degree to which they entail an exploration–exploitation tradeoff (Sutton & Barto, 1998). In both paradigms, every choice is associated with the goals of obtaining a desired outcome (exploitation) or gathering new information about other, perhaps better, outcomes (exploration). In the partial-feedback paradigm, each draw from a payoff distribution contributes to the participant’s earnings (or losses). As a consequence, a balance needs to be struck between the *simultaneous* demands of exploration and exploitation. How people, animals, and even microorganisms balance these simultaneous demands—and how they *should* do it—has been a central issue in reinforcement-learning research (Cohen, McClure, & Yu, 2007; Lee, Zhang, Munro, & Steyvers, 2011; Sutton & Barto, 1998). Various factors have been found to affect this balance. For example, people explore more when the expectation of a change in the payoff structure increases (Cohen et al., 2007) and when the horizon of the task is known to be long (Carstensen, Isaacowitz, & Charles, 1999). Thus far, however, no general optimal solution to this tradeoff has been proposed (but see Gittins, 1979, for optimal tradeoffs in specific cases).

The sampling paradigm separates exploration and exploitation, eliminating the tradeoff between them. The only costs incurred by search are opportunity costs, time, and effort, as in many other information-processing tasks. Although nothing is at stake for the individual during sampling, exploring one payoff distribution more than another may reflect attraction or vigilance toward specific properties of the options. More extensive exploration of a distribution in which losses loom would permit a more accurate evaluation of the quantitative risk of a loss. Against this background, Lejarraga et al. (2012) investigated to what extent the risk of suffering a loss triggers more search than the chance of reaping a gain. Screening published data sets, Lejarraga et al. indeed found evidence for more exploration of distributions that involved the risk of losses, with a relative increase in search of, on average, 25 % (aggregate level) and 29 % (individual level) in the loss relative to the gain domain. Taken together, these results indicate that the choice domain—loss versus gain—has a discernible impact on *exploratory search* in decisions from experience.

One objection to this *loss–gain exploration asymmetry* is that, as pointed out earlier, search itself was not directly incentivized, but only subsequent choice. Therefore, it is unclear to what extent the asymmetry will generalize to situations in which search exacts immediate costs. To address this issue, this study investigates the extent to which the loss–gain search asymmetry generalizes to *costly* search in the partial-feedback paradigm. One proxy of costly exploratory search in this paradigm is the rate of alternation between options: the proportion of times an individual moves from choosing one option to choosing the other option during repeated choices. Hummingbirds, like other animals, are interpreted to be exploiting a patch of flowers when they remain in it—and to be exploring when they move to a different one (e.g., Kramer & Weary, 1991). Similarly, participants can be seen as exploiting an option when they repeat a choice—and as exploring when they switch to a different option.^2^ The rate of alternation is a standard proxy for exploration and has been shown to decay over time in repeated-choice tasks as people move from initial exploration to subsequent exploitation (e.g., Cohen et al., 2007; Gonzalez & Dutt, 2011, 2012; Hills & Hertwig, 2010, 2012; Yechiam, Busemeyer, Stout, & Bechara, 2005). To the extent that the loss–gain exploration asymmetry observed in the sampling paradigm generalizes to costly exploration, one would expect a higher alternation rate (assuming it to be an approximate measure of exploration) in the loss domain than in the gain domain. Is there any evidence for such an asymmetry?

Yechiam, Zahavi, and Arditi (2015) examined this question in a particular type of decision problem, namely, one in which two options offered the same expected value (EV), and the gambles were symmetric, with a 50 % chance of winning (or losing) an amount of money and 50 % of not winning (or losing) anything. They found that, on average, there were higher alternation rates in the loss than in the gain domain. Building on this initial result, we pursued three questions: First, by examining the effect of choice domain on exploratory search at the individual level and in a broad variety of decision problems, we investigated whether and to what extent the asymmetry observed in exploratory search is a robust behavioral regularity. Second, we examined whether differences in exploration at the individual level translate into differences in risk taking in terms of loss aversion. Third, we used cognitive modeling to reveal the mechanisms underlying any differences found in exploratory search as a function of domain.

## Method

Our analysis took advantage of the largest available data set in research employing the partial-feedback paradigm. Specifically, we used the data set collected in the Technion Prediction Tournament (TPT; Erev et al., 2010). The TPT is a prediction competition in which different models were fitted to people’s decisions from experience across 60 problems. The models were then used to predict people’s choices in 60 new problems. The competition involved two decisions-from-experience paradigms: the sampling paradigm (analyzed in Lejarraga et al., 2012) and the partial-feedback paradigm that we consider here. Each of the 120 problems represents a choice between a safe option offering a medium (M) payoff with certainty and a risky option offering a high (H) payoff with some probability (pH), and a low (L) payoff with the complementary probability. In each problem, participants made 100 repeated choices between the risky and the safe option. After each choice, participants received feedback on the payoff they obtained. M, H, pH, and L were generated randomly, and a selection algorithm assured that the 120 problems differed in domain (gains, losses, and mixed payoffs) and probability (high, medium, and low). Each of 200 participants made decisions in a subset of 12 problems: four in the gain domain, four in the loss domain, and four in the mixed domain. The subset of problems was predefined, but participants were assigned to a subset at random. Two participants were excluded because of errors in the payoffs. The final sample consisted of 198 participants.

## Results

### Is there, on average, a loss–gain search asymmetry in the rate of exploration?

**Fig. 1:**
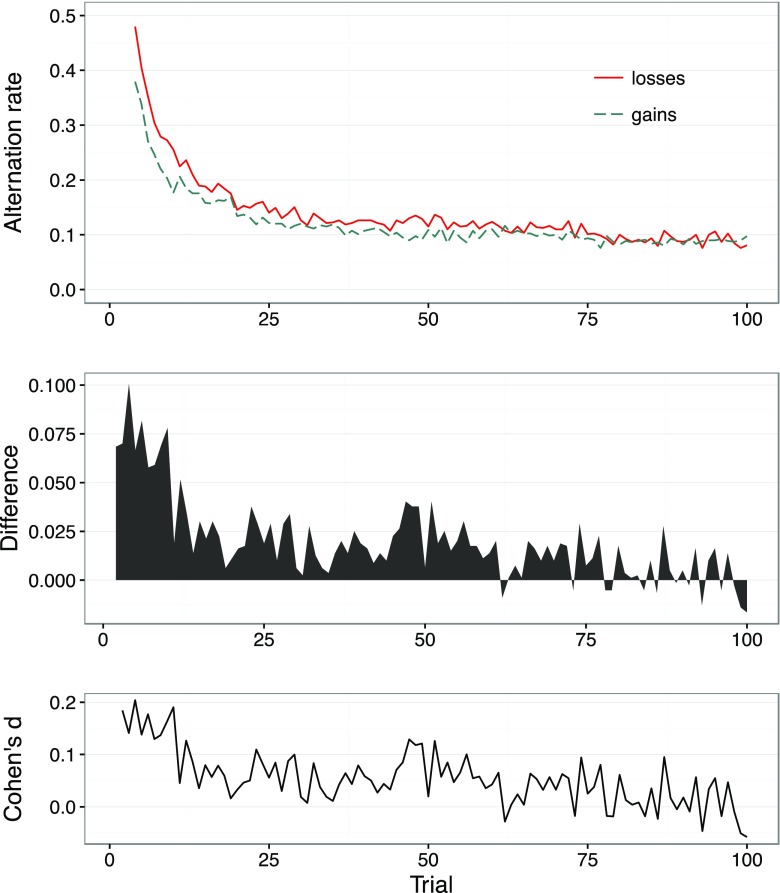
Alternation rate between options (upper panel), separately for the gain and loss domains, across all 100 choice trials for the partial-feedback paradigm of the Technion Prediction Tournament data set (Erev et al., 2010). Difference between the alternation rates in the loss and gain domains (middle panel). Effect size measure, Cohen’s *d*, of the difference between alternation rates (lower panel)

We calculated the mean alternation rate averaged across all participants and all problems. Figure 1 shows the rate across the choice sequences (i.e., for each of the 100 choices made within each problem; upper panel), separately for the gain and loss domains, and the difference between the respective domain-specific rates (middle panel). The results show the typical transition from exploration to exploitation, with a high rate of alternation in early trials, increasingly replaced by exploitation and a lower rate of alternation. Figure 1 (middle panel) also shows a consistent loss–gain exploration asymmetry. Specifically, participants explored more in loss than in gain payoff distributions. This asymmetry is most pronounced in the earlier choices. It then decreases with number of choices, and even reverses at trial 62. Although the difference is small in each individual trial (see Cohen’s *d* values in the lower panel of Fig. 1), individuals’ rate of alternation is higher in the loss domain than in the gain domain in a total of 86 % of the trials, 95 % CI [80 %, 93 %].^3^

### Is there a loss–gain exploration asymmetry in individuals’ rate of exploration?

**Fig. 2:**
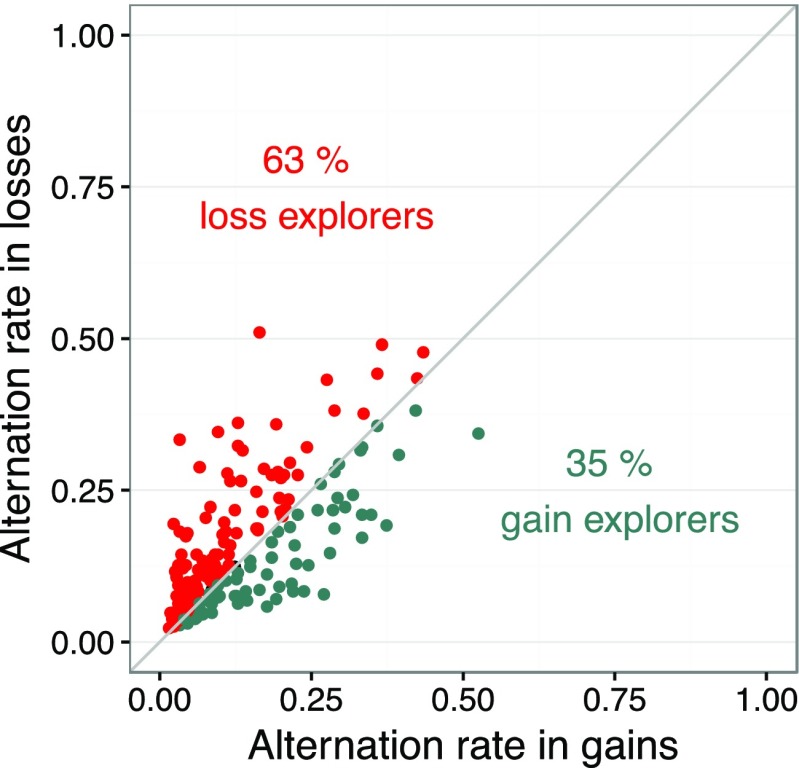
Individuals’ mean alternation rate (averaged across all problems an individual responded to and across all 100 choice trials per problem), separately for the gain and loss domain for the partial-feedback paradigm of the Technion Prediction Tournament data set (Erev et al., 2010)

Figure 1 plots aggregate data. Is the loss–gain search asymmetry also present at the individual level? To answer this question, we calculated the alternation rate for each participant (aggregated across all choices in all problems), separately for the gain and loss domain. Figure 2 plots individuals’ mean alternation rate. A point above the diagonal represents an individual who alternates (explores) more in the loss than in the gain domain. In contrast, a point below the diagonal represents an individual who alternates (explores) more in the gain than in the loss domain. Most participants (63 %) alternated more in the loss domain; 35 % alternated more in the gain domain. That is, almost twice as many people explored more in the loss domain than in the gain domain, 27 % [17 %, 37 %]. Only 2 % showed symmetrical rates (i.e., are located on the diagonal).

### The loss–gain exploration asymmetry: An artifact of the magnitude of EV?

**Fig. 3:**
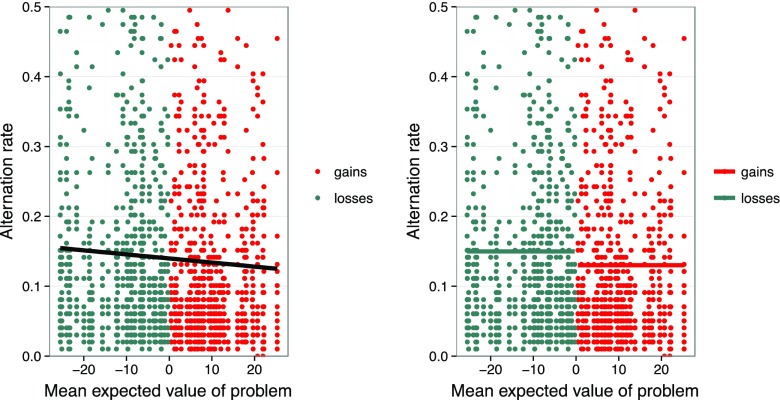
Relationship between alternation rate and magnitude of problems’ EVs in the partial-feedback paradigm of the Technion Prediction Tournament data set (Erev et al., 2010). The graph shows only alternation rates within 0 and .5. The left panel shows a linear model with mean EVs as the only predictor. The right panel shows a model with domain of choice as the only predictor

Options in the loss domain by definition have lower (negative) EVs than do those in the gain domain. It is therefore possible that the loss–gain asymmetry in exploratory search could be a function of the options’ EVs rather than of the domain. Specifically, people may exploit more—and explore less—the higher an option’s EV. To examine this possibility, we calculated the mean EV of each decision problem by averaging its safe option and the EV of its risky option. We then calculated the alternation rate in each problem for each participant. Using the 80 problems in the gain and loss domains of the TPT, we used mixed models to examine the relative impact of mean EV and of domain on alternation rate. First, we fitted a baseline model of alternation rate with random intercepts for each participant. We then fitted a second model, adding mean EV to the baseline model (see Fig. 3, left panel), and a third model, adding the domain of choice to the baseline model (see Fig. 3, right panel). We assessed the significance of the predictors, as well as their relative explanatory power, by using likelihood ratio tests to compare nested models. The relationship between alternation rate and domain was significant, χ^2^(1) = 11.07, *p* < .001, as was the relationship between alternation rate and mean EV, χ^2^(1) = 8.10, *p* = .004. However, domain of choice had higher explanatory power than did mean EV: A model with both mean EV and domain was not better than a model with only domain, χ^2^(1) = 0.02, *p* = .89, but was almost significantly better than a model with only mean EV, χ^2^(1) = 2.99, *p* = .08.

### Does the loss–gain exploration asymmetry result in more loss aversion in choice?

According to Yechiam and Hochman (2013a), the possibility of a loss intensifies the attention that a decision maker devotes to the potential outcomes of a choice, without necessarily triggering loss aversion. *Loss attention* raises the question of to what extent individuals who explore the loss domain more intensively also show increased aversion to losses when choosing. Because the alternation rate is not orthogonal to participants’ choice in the partial-feedback paradigm (the exploration–exploitation dilemma), we used different problems to measure exploration and to examine its relationship to loss aversion. Specifically, we first used problems in the gain and loss domains to classify participants as predominantly “loss explorers” versus predominately “gain explorers.” We then used problems in the mixed domain to analyze the degree of loss aversion, thus also taking advantage of the fact that it is in precisely the mixed domain that loss aversion has mostly been examined (Ert & Erev, 2008, 2013).

**Fig. 4:**
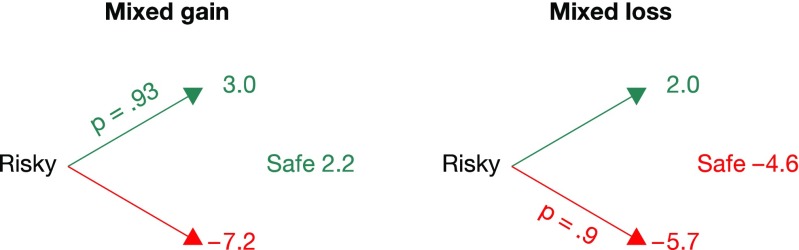
Two illustrative decision problems from the mixed domain used in the partial-feedback paradigm of the Technion Prediction Tournament data set (Erev et al., 2010): a *mixed-gain problem*, in which the safe option is a gain outcome (left), and a *mixed-loss problem*, in which the safe option is a loss outcome (right)

We classified individuals above the diagonal in Fig. 2 as *loss explorers*, and individuals below the diagonal as *gain explorers* (the few individuals with symmetric exploration behavior were omitted from the analysis). We then used all 40 mixed problems to examine whether loss explorers were also more loss averse than gain explorers. There were two classes of decision problems in the mixed domain. As Fig. 4 shows, all mixed problems included gains and losses, but one class offered a safe gain option (henceforth mixed-gain problems), whereas another offered a safe loss option (henceforth mixed-loss problems).

**Fig. 5:**
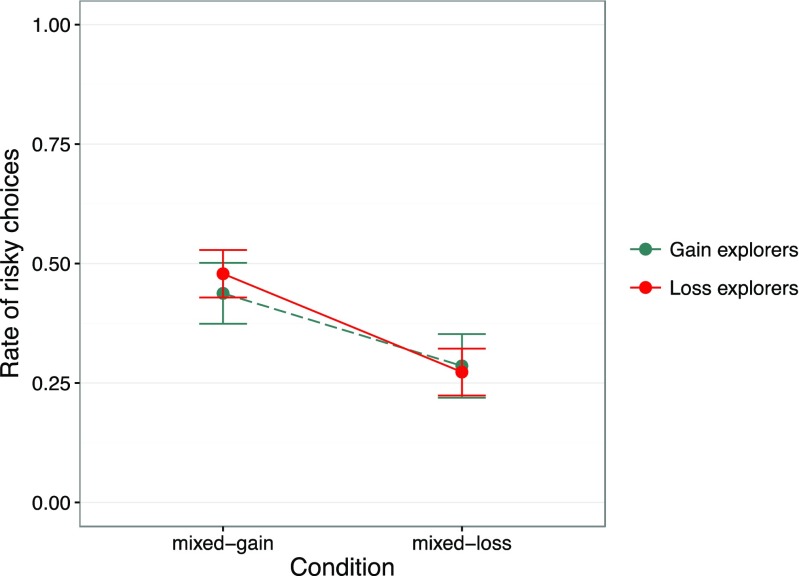
Proportion of choices of the risky payoff distribution, separately for gain and loss explorers (see text) and for mixed-gain and mixed-loss problems (see Fig. 4) in the partial-feedback paradigm of the Technion Prediction Tournament data set (Erev et al., 2010). Error bars show 95 % confidence intervals. Gain explorers are shown by a dashed green line and loss explorers by a solid red line. (Color figure online)

We calculated the proportion of choices of the risky option for loss and gain explorers, separately for mixed-gain and mixed-loss problems. The mixed-gain problems offer people who are averse to losses the choice of a seemingly safe gain.^4^ As Fig. 5 shows, the 95 % confidence intervals of the proportions of risky choices overlapped notably on both mixed-gain and mixed-loss problems, meaning that there was no difference in loss and gain explorers’ choices. More precisely, in mixed-gain problems, the proportions of risky choices from gain and loss explorers were 44 % [37 %, 50 %] and 49 % [43 %, 53 %], respectively, and their difference was –4 % [–12 %, 4 %]. Similarly, in mixed-loss problems the proportions of risky choices from gain and loss explorers were 28 % [22 %, 35 %] and 27 % [22 %, 32 %], respectively, and their difference was 1 % [–6 %, 9 %].

These results suggest that individuals who allocate more effort to exploring the loss domain than the gain domain do not avoid losses in choice. That is, the loss–gain exploration asymmetry does not predict or translate into loss aversion in choice. This finding is consistent with our previous result showing that increased search in the loss domain (relative to the gain domain) in the sampling paradigm does not produce a larger description–experience gap in choices (Lejarraga et al., 2012). The exploration asymmetry thus appears to be a behavioral regularity that cannot simply be reduced to loss aversion or to being a close analog of loss aversion. To better understand its essence, we next applied cognitive modeling to shed light on the processes underlying exploration asymmetry.

## Models of choices

To further examine the mechanisms behind the observed asymmetry in exploratory search, we first identified plausible models of behavior in the partial-feedback paradigm, including models that do and do not assume loss aversion. Second, we fitted each model to the choice behavior of each individual, calibrating the parameters so as to minimize the discrepancy between the prediction of the model and the actual choice observed in each trial. Third, we examined how the model predictions tracked the observed asymmetry in exploration; that is, we evaluated the extent to which, aggregated across individuals, the model predictions were able to reproduce the upper panel in Fig. 1.

We entered three types of models into the analysis: (i) four specifications of reinforcement-learning models^5^; (ii) an instance-based learning model, which has previously been shown to capture aggregate behavior in this data set accurately (Lejarraga, Dutt, & Gonzalez, 2012),^6^ and (iii) a win-stay-lose-shift model, which explained the behavior of the majority of participants in a similar task (i.e., the Iowa gambling task; Worthy, Hawthorne, & Otto, 2013). We asked three questions: (1) Is the assumption of loss aversion *necessary* to describe the data well? To answer this question, we examined whether models assuming loss aversion fitted the data better than did models *not* assuming loss aversion. (2) Does loss aversion reproduce the observed loss–gain asymmetry in exploration? Here, we identified those participants who were best fitted by parameters indicating loss aversion. We then aggregated model predictions across participants and examined whether the predictions reproduced the loss–gain exploration asymmetry. Finally, (3) which process reproduces the observed asymmetry in exploration?

### Reinforcement-learning models

Reinforcement-learning models have three elements: (a) a utility function that is used to evaluate the outcomes encountered; (b) a learning rule that is used to update the value or expectancy of each option; and (c) a choice rule that is used to select between the options. We analyzed the performance of four reinforcement models that differed in their utility function.

### RL-baseline model

This model assumes that the utility of an outcome is equal to its monetary amount and, importantly, it makes no distinction between gains and losses. The utility *u* of an option for trial *t* is

1 $$ u(t)=x(t), $$

where *x*(*t*) denotes the amount of money won or lost on trial *t*. The model assumes that the decision maker develops an *expectancy* for each option (i.e., an expectation of the utility of the option). The expectancy *E*_*j*_(*t*) for option *j* in trial *t* is

2 $$ {E}_j(t)={E}_j\left(t-1\right)+\phi \cdot {\delta}_j(t)\cdot \left[u(t)-{E}_j\left(t-1\right)\right], $$

where *δ*_*j*_(*t*) = 1 if the outcome of option *j* was observed in trial *t*, and *δ*_*j*_(*t*) = 0, otherwise. The learning rate *ϕ* (0 ≤ *ϕ* ≤ 1) indicates the degree to which the latest option influences the expectancy for the option. Higher *ϕ* values indicate more recency. In the first trial, *E*_*j*_(*t* − 1) is assumed to be 0. Finally, the probability of choosing option *j* follows Luce’s rule (1959) and is defined as follows:

3 $$ P\left(j,t+1\right)=\frac{e^{\theta \cdot {E}_j(t)}}{{\displaystyle {\sum}_i^2}{e}^{\theta \cdot {E}_i(t)}}, $$

4 $$ \mathrm{with}\kern0.24em \theta (t)={\left(\frac{t}{10}\right)}^c, $$

where *θ*(*t*) is the trial-dependent sensitivity to the differences in expectancies and *c* (− 5 ≤ *c* ≤ 5) is a consistency parameter. Positive values of *c* indicate that the option with the higher expectancy will be chosen as the number of trials increases. Negative values of *c* indicate more random choices as the number of trials increases.

This model has been used to capture learning processes in various domains, particularly in repeated choice (Busemeyer & Myung, 1992; Worthy & Maddox, 2014; Yechiam & Busemeyer, 2005).

### RL-lambda model

This model is identical to the RL-baseline model, except that it assumes loss aversion. Therefore, the utility function is not [1] but

5 $$ u(t)=\left\{\begin{array}{ll}x(t)\hfill & if\kern0.24em x(t)\ge 0\hfill \\ {}-\lambda \left|x(t)\right|\hfill & if\kern0.24em x(t)<0\hfill \end{array}\right.. $$

The parameter *λ* (0 ≤ *λ* ≤ 5) indicates the degree of loss aversion. Values above 1 indicate greater sensitivity to losses than to gains, and values below 1 indicate the opposite.

### RL-PVL model

This model is identical to the RL-baseline model, except that it assumes a prospect theory value function (Tversky & Kahneman, 1992; prospect valence learning was proposed by Ahn, Busemeyer, Wagenmakers, & Stout, 2008). Instead of [1], outcomes are valued according to

6 $$ u(t)=\left\{\begin{array}{ll}x{(t)}^{\alpha}\hfill & if\;x(t)\ge 0\hfill \\ {}-\lambda {\left|x(t)\right|}^{\alpha}\hfill & if\;x(t)<0\hfill \end{array}\right.. $$

Parameter *λ* operates in the same manner as in [5]. The shape of the utility function is governed by *α* (0 ≤ *α* ≤ 1), with lower values indicating higher curvature and with *α* = 1 indicating linear utilities.

### RL-EVL model

This model was proposed by Busemeyer and Stout (2002) and named the expectancy valence learning model. It is identical to the RL-baseline model, except that it assumes an alternative specification of loss aversion. Here, the utility of an outcome is

7 $$ u(t)=\left\{\begin{array}{ll}\left(2-w\right)\cdot x(t)\hfill & if\;x(t)\ge 0\hfill \\ {}-w\left|x(t)\right|\hfill & if\;x(t)<0\hfill \end{array}\right.. $$

The *w* (0 ≤ *w* ≤ 2) parameter indicates the weight of losses relative to gains. When *w* = 1, losses and gains are weighed equally; *w* > 1 indicates greater weight to losses than to gains; and *w* < 1 indicates the opposite.

### Win-stay-lose-shift

Alternatively, people may apply a simple heuristic to choose between options in the partial-feedback paradigm (Novak & Sigmund, 1993). According to the win-stay-lose-shift (WSLS) model, people tend to switch to a different option (shift) after experiencing a loss and to replicate a choice (stay) after experiencing a gain. Consistent with the common implementation of this heuristic (Worthy & Maddox, 2014), losses and gains are defined relative to the immediately previous outcome: If the current outcome is higher than or equal to the previous outcome, the trial is considered a win; otherwise, it is considered a loss.

The model makes probabilistic predictions, with two parameters indicating the probability of staying given a win, *p*(*stay*|*win*), and the probability of shifting given a loss, *p*(*shift*|*loss*). Consequently, the corresponding probability of shifting after a win is 1 − *p*(*stay*|*win*) and that of staying after a loss is 1 − *p*(*shift*|*loss*). Because these probabilities are constant across trials, Worthy and Maddox (2014) proposed a process by which they are permitted to change across trials, thus capturing the observation that the reaction to losses and gains changes across trials.

If *x*(*t*) ≥ *x*(*t* − 1), the trial is considered a “win,” and the probabilities for the subsequent trial are

8 $$ p{\left( stay\Big| win\operatorname{}\right)}_{t+1}=p{\left( stay\Big| win\operatorname{}\right)}_t+{\theta}_{p\left( stay| win\right)}\cdot \left(1-p{\left( stay| win\right)}_t\right) $$

9 $$ \mathrm{and}\ p{\left( shift\left| loss\right.\right)}_{t+1}=\left(1-{\theta}_{p\left( shift\left| loss\right.\right)}\right) \cdot p{\left( shift\left| loss\right.\right)}_t, $$

where *θ*_*p*(*stay*|*win*)_ determines the change in *p*(*stay*|*win*) and *θ*_*p*(*shift*|*loss*)_ determines the change in *p*(*shift*|*loss*).

If *x*(*t*) < *x*(*t* − 1), the trial is considered a “loss,” and the probabilities for the subsequent trial are

10 $$ p{\left( shift\left| loss\right.\right)}_{t+1}=p{\left( shift\left| loss\right.\right)}_t+{\theta}_{p\left( shift\left| loss\right.\right)}\cdot \left(1-p{\left( shift\left| loss\right.\right)}_t\right) $$

11 $$ \mathrm{and}\kern0.24em p{\left( stay\left| win\right.\right)}_{t+1}=\left(1-{\theta}_{p\left( stay\left| win\right.\right)}\right) \cdot p{\left( stay\left| win\right.\right)}_t. $$

### Estimation of the models

We estimated each model so as to minimize the difference between its predictions and actual behavior. Specifically, we estimated the parameters of each model for each participant, based on the fit between the model prediction for *t* + 1 to the observed choice, using the log likelihood method (LL). For each model, we calculated the Bayesian information criterion (BIC; Schwarz, 1978):

12 $$ BIC = -2\left(L{L}_{model}\right)+k\cdot \ln (N), $$

where *k* is the number of free parameters estimated in the model and *N* is the number of trials used to calculate the LL of the model (12 problems × 100 trials, *N* = 1, 200).

We used a grid search with .1 increments along the parameter space. When the resulting grid was too large (as in RL-lambda, RL-EVL, and RL-PVL), we used a combination of grid search (with .25 increments) and the Broyden–Fletcher–Goldfarb–Shanno (BFGS) optimization algorithm. All models and estimation routines were written in R and are available in the supplementary material.

## Modeling results

### Is the assumption of loss aversion necessary to describe the data well?

Table 1 shows the accuracy of the models. Lower BIC values indicate better fit. Different specifications of the utility function (RL-lambda, RL-PVL, and RL-EVL) improved the fit of the reinforcement-learning models over the RL-baseline model. However, the resulting parameters did not indicate loss aversion, but the contrary: *λ* and *w* parameters *below* 1 indicate higher sensitivity to gains than to losses. These results support our previous observation that more exploration in the loss domain does not result in choices that reflect aversion to losses. They are also consistent with the idea that losses increase arousal and on-task attention (Yechiam & Hochman, 2013a).

**Table 1:** Mean BIC Scores and Model Parameters

	BIC		*ϕ*		*c*		*λ*		*α*		*W*	
Chance	1,663	(0)	–	–	–	–	–	–	–	–	–	–
RL-baseline	1,401	(334)	0.54	(0.41)	–0.92	(2.93)	–	–	–	–	–	–
RL-lambda	1,253	(318)	0.40	(0.40)	0.24	(2.10)	0.88	(1.69)	–	–	–	–
RL-PVL	1,195	(291)	0.41	(0.37)	1.43	(1.39)	0.98	(1.83)	0.50	(0.36)	–	–
RL-EVL	1,267	(329)	0.52	(0.40)	0.32	(2.25)	–	–	–	–	0.64	(0.8)
	BIC		*p*(*stay*|*win*)		*p*(*shift*|*loss*)		*θ*_*p*(*shift*|*loss*)_		*θ*_*p*(*stay*|*win*)_			
WSLS	723	(324)	0.58	(0.30)	0.63	(0.31)	0.21	(0.22)	0.25	(0.26)		

*Note*. Values in parentheses are standard deviations. The RL-lambda, RL-PVL, and RL-EVL models include loss aversion parameters (*λ* and *W*). Values larger than 1 on these parameters would indicate loss aversion

### Does loss aversion reproduce the observed loss–gain asymmetry in exploration?

**Fig. 6:**
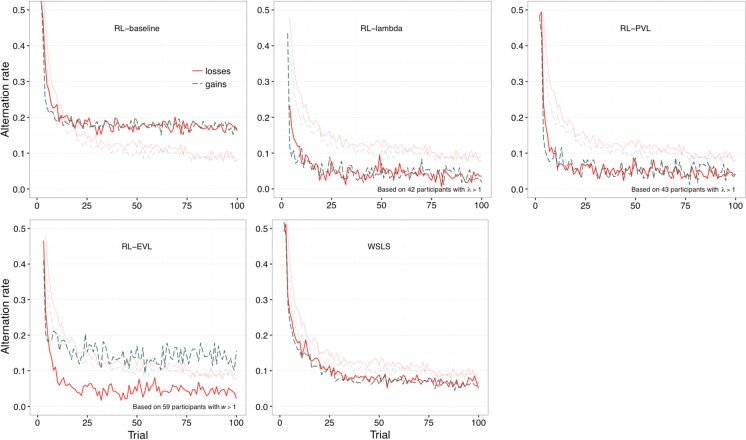
Alternation rates by trial predicted by the best-fitting models for each individual. The darker lines indicate alternation rates predicted by the models. The lighter lines indicate the observed alternation rates

To address this question, we focused on participants whose best-fitting parameters indicated loss aversion (*λ* > 1 or *w* > 1). We obtained the models’ predictions for each individual and recorded the alternations in each trial. We then calculated the alternation rate across participants for each trial in order to examine whether the model predictions reproduced the loss–gain asymmetry in exploration. As Fig. 6 shows, models with loss aversion—the RL-lambda, the RL-PVL, and RL-EVL model—did not reproduce the observed asymmetry in exploration (displayed in lighter colors). In the RL-lambda and RL-PVL models, the alternation rates in the gain and loss domains are indistinguishable, whereas the RL-EVL model shows more exploration in the gain than in the loss domain. These findings suggest that the observed asymmetry in exploration does not result from assuming different utilities for losses and gains, but from a different process.

### Which process reproduces the observed asymmetry in exploration?

We found strong evidence that participants employ a win-stay-lose-shift heuristic, and, importantly, that this applies when losses and gains are defined relatively and not in absolute terms. Because the model assumes that participants repeat their choice after a win but switch after a loss, it implies that reactions to gains versus losses differ, with losses generating higher alternation rates. Indeed, the best-fitting parameters in Table 1 indicate a higher tendency to switch after a loss (.63) than after a gain (.42 = 1 – .58). However, the definition of a gain and a loss in this model does not depend on the positive (gain) or negative (loss) sign of the outcome, but on the relative magnitude of current and previous outcomes. Even with this conceptualization of gains and losses, the model captures three characteristics of the observed patterns of exploration (see Fig. 1, upper panel): (1) a higher exploration rate among losses than among gains in approximately the first 50 trials (Fig. 6), (2) a gradual transition from exploration to exploitation, and (3) and a converging rate of exploration across domains that approaches 0.1.

## Why does the asymmetry in exploration emerge?

The results of our modeling point to a possible process by which people end up exploring options more under the threat of losses than under the promise of gains. It thus suggests *how* the observed asymmetry in exploration could emerge but not *why* it emerges. Next, we discuss two possible explanations for the asymmetry. Let us first highlight that in decisions from experience—such as those studied here—people face uncertainty (or ambiguity, a term more commonly used in the economics literature). Specifically, they do not initially know either the outcome space they face or the outcomes’ probabilities. Exploration is the process by which they learn about the outcome space and relative frequencies, thus reducing uncertainty. It has often been observed that people facing uncertainty tend to employ simple heuristics and rules that enable choices and inferences, even when knowledge of likelihoods is nonexistent or relatively rudimentary (Gigerenzer, Hertwig, & Pachur, 2011; Savage, 1954). Under uncertainty, some simple processes may even be prescribed: “Such rules as minimaxing . . . are usually prescribed for situations of ‘complete ignorance,’ in which a decision-maker lacks any information whatever on relative likelihoods” (Ellsberg, 1961, p. 657). Building on the premise of simple processes being engaged to tame uncertainty, we suggest that the asymmetry in exploration could be a by-product of the reliance on boundedly rational processes of human decision making. Specifically, we consider Simon’s (1956) notion of *satisficing* and Wald’s (1945) *minimax* rule.

### Aspiration levels as stopping rules

Simon (1956) offered a novel view on rationality, according to which organisms make good enough (“satisficing”) rather than optimizing choices, particularly under circumstances in which the conditions for rationality postulated by the model of neoclassical economics are not met (e.g., full knowledge of the probability distributions for uncertain events; Simon, 1989, p. 377) and the optimum is not computable. His concern was with how decision makers can find satisfactory solutions in a realistic world. Let us consider the principle of satisficing in the context of Simon’s model of a simple toy organism whose sole goal is to eat. It has limited storage capacity and needs to maintain a minimum level of energy to survive. Once this level of “aspiration” is surpassed, the organism rests. To meet its energetic needs, the organism explores the environment (e.g., a plane with heaps of food or a maze with branching paths) to locate food and then eats (exploits) it.

How could a gain–loss exploration asymmetry arise for this simple organism? Let us first consider what counts as a loss domain. One construal could be in terms of the organism’s energetic state. From this perspective, the organism will enter the loss domain whenever its energy level falls below a subsistence threshold—its aspiration level. As long as the organism is in this state, it needs to explore. Once the energy level is back above this threshold and the organism has thus entered the gain domain, it will cease to explore. An alternative construal could be not in terms of the organism’s state but in terms of the ecology it faces. For instance, if the quality of food patches is such that each single patch does not suffice to push the organism above the subsistence threshold, or if good-enough patches are extremely rare, the organism is in the loss domain. Consequently, it will need to keep exploring once a patch has been exploited (or once the rate of return has dropped below the average rate of payoff for the entire area; Charnov, 1976). In contrast, a resources-rich environment (gain domain) will offer many patches that yield “enough.” Finding a dense patch will thus render it unnecessary to explore further. Still another construal defines the loss domain not in terms of resources per se but in terms of competitive dynamics. A loss domain could then be one in which patches come with substantial predation risks. In this context, an organism may have to continue exploring until it finds a relatively “safe” patch. The gain domain would be one in which the predation risk is small or nonexistent.

Admittedly, these are simplistic and hypothetical scenarios and interpretations of what loss versus gain domain could mean. Yet in each one, the toy organism proposed by Simon would explore more in the loss than in the gain domain. To the extent that the experimental decisions-from-experience paradigms are (remote) proxies of these scenarios, the activation of aspiration levels that function as stopping rules may produce asymmetries in exploratory behavior. Interestingly, the win-stay-lose-shift model in our analysis can be viewed as a kind of satisficing model, in which the decision maker uses an aspiration level that resets after every outcome and makes a probabilistic choice.

### The minimax rule

Another simple process that could give rise to the observed asymmetry in exploration is the *minimax* rule, a choice rule devised by Wald (1945; see also Savage’s, 1954, *maximin* rule) to “minimize the maximum risk” when facing uncertain options. According to minimax, the decision maker ranks the options according to his or her worst-case outcome and chooses the one with the least worst outcome. Minimax can be seen as making a bet about the structure of the environment and acts as if it “lives” in a hostile environment where the worst thing that could happen will always happen—and could potentially pose an existential threat.^7^ The rule implies that the decision maker searches for the worst possible outcome for each option. It is only after exploration has revealed all bad things that could possibly happen that the decision maker can hope to choose in a way that maximizes the minimum outcome. If the same decision maker encounters positive outcomes, which no longer trigger the goal of preventing the worst, one may expect explorative efforts to be less exhaustive than in the actual loss domain.

The two possible explanations for why a loss–gain asymmetry may occur are admittedly speculative. Yet they offer a link from research on decisions from experience to Simon’s (1956, 1978) concepts of satisficing and aspiration level as well as to Wald’s (1945) and Savage’s (1954) work on strategies for making decisions in the face of uncertainty. Both explanations, combined with the specific win-stay-lose-shift account proposed here, can inform future work on the dynamic of exploration across the various decisions-from-experience paradigms.

## Conclusions

In most investigations of loss aversion, cumulative prospect theory’s loss-aversion parameter has been gauged by fitting it to individuals’ choices. Yet choice is not the only observable manifestation of humans’ (assumed) preferences. External search for information is another. Research on decisions from experience (see Hertwig & Erev, 2009) has taken advantage of this search behavior to advance the scientific understanding of human choice. Analyzing exploratory search in the sampling paradigm, Lejarraga et al. (2012) found a loss–gain exploration asymmetry: When facing the risk of losses, most people invested more effort in exploring potentially disadvantageous (loss) than advantageous (gain) payoff distributions. However, this asymmetry occurred in a situation in which exploration did not have explicit monetary consequences; the sampling paradigm incentivizes only choice, but not search.

To find out whether the loss–gain exploration asymmetry generalizes to costly search, we analyzed data collected within another decision-from-experience paradigm: the partial-feedback paradigm. Unlike the sampling paradigm, this paradigm invokes an exploration–exploitation trade-off: The outcome of each draw made increases or decreases the final payoff. Nevertheless, we found the same kind of loss–gain exploration asymmetry as in the sampling paradigm (Lejarraga et al., 2012). In other words, in both *costly and cost-free search*, most individuals explored more when facing the threat of losses than the promise of gains. This finding is consistent with the notion of loss attention, that is, intensified vigilance in the face of potential losses (Yechiam & Hochman, 2013a, 2013b, 2014). Interestingly, however, this robust loss–gain exploration asymmetry is not a *precursor* of loss aversion in choice. In our analysis, loss explorers ended up making the same choices as gain explorers in the domain of mixed gambles.

We then used cognitive modeling to investigate the mechanism behind the loss–gain exploration asymmetry. Specifically, we fitted and analyzed the predictions of various models that assumed different instantiations of utility, including linear utilities, different forms of loss aversion, and no utility function at all. Three patterns emerged from the modeling analysis. First, loss aversion was not manifest in the parameter values of models that were fitted to individuals’ choices in the partial-feedback paradigm (Table 1). Second, models equipped with a parameter to capture loss aversion did not fit the data better than a win-stay-lose-shift heuristic (Table 1). In addition, the former models could not reproduce the asymmetric pattern of exploration, whereas the WSLS model captured both the asymmetry (Fig. 6) and the overall level of exploration. Importantly, it also captured the reduction in the asymmetry across trials: The model incorporates two parameters (*θ*s) that gradually change the initial tendencies to stay after a win and to switch after a loss, prompting these tendencies to converge to 1 and 0, respectively. Therefore, the tendency to switch after a loss or after a gain reduces to 0 across trials, grinding away the differences in exploration in gains and losses (as conceptualized in the model).

The assumed human aversion to losses—that is, the stronger weighting of losses relative to gains—has nearly a law-like status in psychology and beyond. According to Wakker (2010), “the main empirical phenomenon concerning the distinction between gains and losses is loss aversion” (p. 238). Kahneman, whose work with Tversky on prospect theory established loss aversion as a key concept (though it was invoked earlier; e.g., Robertson, 1915, p. 135), recently emphasized its importance beyond the laboratory and beyond student samples in an interview:

In my classes, I say: “I’m going to toss a coin, and if it’s tails, you lose $10. How much would you have to gain on winning in order for this gamble to be acceptable to you?” People want more than $20 before it is acceptable. And now I’ve been doing the same thing with executives or very rich people, asking about tossing a coin and losing $10,000 if it’s tails. And they want $20,000 before they’ll take the gamble. (Richards, 2013, para. 6)

For decades, the existence and the magnitude of loss aversion has mostly been inferred from such overt choices based on stated probabilities. It is only recently that researchers have begun to look at lower-level processes and discovered that loss aversion is not the only mechanism at play. The threat of losses also triggers heightened autonomic responses such as physiological arousal (Hochman & Yechiam, 2011; Yechiam, Retzer, et al., 2015). Equally important, this recent research has shown that there can even be a dissociation between increased arousal and attention and behavioral loss aversion in experienced-based decision tasks: An increased focus on the task in response to the threat of losses may enhance the individual’s sensitivity to the task’s reinforcement structure but does not necessarily translate into behavioral loss aversion, that is, to people giving more weight to losses than to gains when making decisions.

Another behavioral dimension in which increased attention to the payoff structure of a task can become manifest is search. Like Yechiam and Hochman (2013a, 2013b, 2014), we found more exploration in the loss than in the gain domain, as well as a dissociation between this exploratory behavior and loss aversion in experienced-based choice (see also Lejarraga et al., 2012). Building on Yechiam and Hochman’s findings, we first showed that this asymmetric exploration pattern is prevalent both at the aggregate and at the individual level, and that it also emerges when a large variety of problems is used. Second, we found that the decisions made by individuals who show this asymmetry do not differ qualitatively from those of individuals who show the opposite asymmetry. Third, and importantly, we identified a likely candidate mechanism behind the observed loss–gain asymmetry in exploration: the win-stay-lose-shift heuristic. This mechanism does not invoke loss aversion but postulates that losses lead to a lower propensity to replicate a choice than gains do.

Taken together, our results indicate that the loss–gain asymmetry in exploration is a robust behavioral regularity in contexts where people have to learn from experience when making choices (see also Ert & Erev, 2008, 2013; Walasek & Stewart, 2015) and that it is an invariant property of both cost-free and costly search. In addition, in both kinds of search, there seems to be a dissociation between loss–gain asymmetry in exploration and loss aversion in choice.

## Electronic supplementary material

Below is the link to the electronic supplementary material.

ESM 1 (DOCX 15 kb)

## Footnotes

1 Of course, there are several sophisticated methods to track information search in description-based paradigms (see Schulte-Mecklenbeck, Kühberger, & Ranyard, 2011), such as eye-tracking devices (Glöckner, Fiedler, Hochman, Ayal, & Hilbig, 2012). These methods track observable proxies of the decision process, and on that basis make inferences about other aspects that are not directly observable.

2 In a recent review of the literature on the exploration–exploitation tradeoff (Mehlhorn et al., 2015), a set of experts endorsed the following view: “Behavior is interpreted as exploration if it alternates between patches or options, is unfocused, and is variable over time. Behavior is interpreted as exploitation if it remains within a patch or option, is focused, and is stable over time” (p. 193). There is one important difference between our context and the animal foraging literature, however. In the partial-feedback paradigm, a payoff distribution—unlike a food patch—commonly does not deplete (because payoffs are drawn *with replacement*).

3 The 95 % confidence interval does not include 50 %, meaning that we are 95 % confident that the hypothesized asymmetry will generalize to other experiments. In the rest of this Results section, we use the “new statistics” to analyze results (Cumming, 2012). We report means and 95 % confidence intervals and, when appropriate, we use differences in means as measures of effect size.

4 To derive stronger, directional, predictions, we also compared the predictions of an EV model with those of the same model including a loss-aversion parameter set at 2.25 (Tversky & Kahneman, 1992). Loss aversion increases the attractiveness of the safe option in both mixed-gain and mixed-loss problems. Moreover, this preference for the safe option should be higher in mixed-gain than in mixed-loss problems. Therefore, if loss explorers are more averse to losses, they should reveal a lower preference for the risky option than gain explorers in both types of problems, and this difference should be larger in the mixed-gain problems. As Figure 5 shows, there was no such difference.

5 The four reinforcement-learning models we implemented represent a subset of the wide variety of reinforcement-learning models that have been proposed. We assumed relatively simple processes in our analysis to examine the influence of loss aversion as orthogonally as possible from other processes. For example, Worthy, Pang, and Byrne (2013) proposed an eight-parameter model incorporating a reinforcement-learning mode and a perseveration mode. Such a model could potentially capture behavior well, but its predictions are derived from a weighted average of a standard reinforcement process and a win-stay-lose-shift process, making the interpretation of results more intricate than when those processes are compared separately, as is done here.

6 To put this model on the same footing as the other models, we fitted it at the individual level. This resulted in parameters that were unstable across estimation runs. We therefore do not detail the model and its results—the code is available in the supplementary material.

7 Recent neuroscientific evidence suggests that such a pessimistic outlook may be more likely to be triggered in a world ripe with uncertainty: Under uncertainty, the brain is alerted to the fact that information is missing, that choices based on the information available therefore carry more unknown (and potentially dangerous) consequences, and that cognitive and behavioral resources must be mobilized in order to seek out additional information from the environment. (Hsu, Bhatt, Adolphs, Tranel, & Camerer, 2005, p. 1683)
