# Supplementary material for: How the threat of losses makes people explore more than the promise of gains
Source: Psychon Bull Rev. 2016 Sep 12;24(3):708–20. doi: 10.3758/s13423-016-1158-7 (PMC5486855; doi:10.3758/s13423-016-1158-7)
Supplement: Supplementary file 1 — (DOCX 15 kb) [file 13423_2016_1158_MOESM1_ESM.docx]

Supplementary Material

Median parameter estimates of models

|  | $\phi$ | *c* | $\lambda$ | $\alpha$ | *W* |
| --- | --- | --- | --- | --- | --- |
| RL-baseline | 0.60 | -0.30 | ‒ | ‒ | ‒ |
| RL-lambda | 0.23 | 0.19 | 0.00 | ‒ | ‒ |
| RL-PVL | 0.24 | 1.01 | 0.00 | 0.47 | ‒ |
| RL-EVL | 0.50 | 0.16 | ‒ | ‒ | 0.12 |
|  | $p(stay\vert win)$ | $p(shift\vert loss)$ | $\theta_{p\left( shift \vert loss \right)}$ | $\theta_{p\left( stay \vert win \right)}$ |  |
| WSLS | 0.70 | 0.60 | 0.10 | 0.10 |  |
